# Editorial

**Published:** 1904-03-15

**Authors:** 


### EDITORIAL.

THE DAILY MEDICAL is the title of a new enterprise published every day in New York City. It is a quarto sheet with a page ten by fifteen inches, some issues being double. It is edited by M. W. Curran, M.D., and published by the Medical Publishing Co., of America, at the rate of one dollar per year. Its work is not only to gather news of every variety of medical activity or interest, but each issue contains one or more original articles by eminent practitioners or investigators. The editorial page is varied and well conducted. If we were to make a suggestion it would be that the editorials be made shorter, although we realize that this is difficult because of the nature of the material. A bright comment on a live topic will be read if it can be condensed to a single paragraph when the same topic exhaustively treated will not be read. We wish the enterprise success.

THE SOUTHERN BRANCH OF THE NATIONAL DENTAL ASSOCIATION met at Washington, February 23d to 25th, with a program of twenty-seven papers and eighteen clinics. We suppose the more participants the better the meeting should be, on the theory that every one that takes a part will be pleased with the meeting anyway. In our judgment, it is a mistake to crowd from three to four papers into every session of a three days' meeting. Any one who will give close attention to so many papers and their discussions will be so exhausted physically and overfed mentally that no real good can come from such a process of cramming. On this occasion there was some relief by social events including a reception by President Roosevelt and a banquet at the hotel.

ATTENDANTS AT STATE SOCIETY MEETINGS.—The *Digest* prints an editorial on methods of increasing attendance at our State meetings. The remedy suggested is that the state meetings are not advertised as they should be, and that programs ought to be gotten up six months in advance. These suggestions as well as many others have been tried, expecting to strike a popular chord which would just force not only the five percent but at least fifty percent of the dentists in every State to attend these meetings. We believe a good program should have only a few good papers on such topics as are in the minds of men in practice, and as large a number of clinics as can be provided. The clinics, as the papers, should be selected with great care. The rank and file of the profession do not care to spend three days of time and considerable money to hear long papers and long-drawn-out discussions. A program that is promising, supplemented by individual solicitation, will bring large results. Another point also is that the program should be home-made and not foreign-born. It is not worth while to say to a practitioner that he ought to attend and support the State society; he knows his own duty as well as anyone else and will resent being drummed. A good program and a cordial and fraternal invitation ought to bring out every man who desires to improve himself.

—✦—

THE COMMERCIAL EXHIBITOR AS A CLINICIAN.—The *Digest* in its February issue touches up the dentist who has invented some appliance or facility which he endeavors to exploit commercially throughout the dental society clinic, claiming that he is undignified and to be suppressed on every occasion. It may be undignified and even unethical to invite or permit such exhibitions, but the truth is that very often these clinics are the most interesting ones on the program and attract the crowd. Naturally, a man who has spent time and thought in devising a machine or a process that is sufficiently practical to warrant him in providing it for others, is enthusiastic about it, and can give a very attractive exhibit or clinic with it, and its instructive value seems to justify the executive officers in permitting him to present it in the clinics. Then again these clinics are generally practical and serve to attract many men to society meetings who would not go for the literary program alone. This is a well-recognized fact by every one who has the duty of making his clinic as attractive as possible and he is always greatly tempted to allow clinics which he perhaps might not wish if he were to allow his professional ideals to control him. It is a matter that should be handled with great wisdom. If the lines are too rigidly drawn we should lose many interesting and valuable demonstrations. Some men who invent good things which they have placed on the market through the regular supply houses, would not care to exhibit themselves as tradesmen or at least in advertising their own inventions, or appear to be soliciting trade for them. We do not think any executive officer with the higher professional instincts will fail to differentiate the chronic self-seeker from the benevolent professional enthusiast.

